# Cerebral ischemia induces microvascular pro-inflammatory cytokine expression via the MEK/ERK pathway

**DOI:** 10.1186/1742-2094-7-14

**Published:** 2010-02-26

**Authors:** Aida Maddahi, Lars Edvinsson

**Affiliations:** 1 Division of Vascular Research, BMC, Lund University, Lund, Sweden; 2 Department of Internal Medicine, Institute of Clinical Sciences, Lund University, Lund, Sweden

## Abstract

**Background:**

Cerebral ischemia from middle cerebral artery wall (MCA) occlusion results in increased expression of cerebrovascular endothelin and angiotensin receptors and activation of the mitogen-activated protein kinase (MAPK) pathway, as well as reduced local cerebral blood flow and increased levels of pro-inflammatory mediators in the infarct region. In this study, we hypothesised that inhibition of the cerebrovascular inflammatory reaction with a specific MEK1/2 inhibitor (U0126) to block transcription or a combined receptor blockade would reduce infarct size and improve neurological score.

**Methods:**

Rats were subjected to a 2-hours middle cerebral artery occlusion (MCAO) followed by reperfusion for 48 hours. Two groups of treated animals were studied; (i) one group received intraperitoneal administration of a specific MEK1/2 inhibitor (U0126) starting at 0, 6, or 12 hours after the occlusion, and (ii) a second group received two specific receptor antagonists (a combination of the angiotensin AT_1 _receptor inhibitor Candesartan and the endothelin ET_A _receptor antagonist ZD1611), given immediately after occlusion. The middle cerebral arteries, microvessels and brain tissue were harvested; and the expressions of tumor necrosis factor-α (TNF-α), interleukin-1ß (IL-1ß), interleukin-6 (IL-6), inducible nitric oxide synthase (iNOS) and phosphorylated ERK1/2, p38 and JNK were analysed using immunohistochemistry.

**Results:**

We observed an infarct volume of 25 ± 2% of total brain volume, and reduced neurological function 2 days after MCAO followed by 48 hours of recirculation. Immunohistochemistry revealed enhanced expression of TNF-α, IL-1ß, IL-6 and iNOS, as well as elevated levels of phosphorylated ERK1/2 in smooth muscle cells of ischemic MCA and in associated intracerebral microvessels. U0126, given intraperitoneal at zero or 6 hours after the ischemic event, but not at 12 hours, reduced the infarct volume (11.7 ± 2% and 15 ± 3%, respectively), normalized pERK1/2, and prevented elevation of the expressions of TNF-α IL-1ß, IL-6 and iNOS. Combined inhibition of angiotensin AT_1 _and endothelin ET_A _receptors decreased the volume of brain damaged (12.3 ± 3; *P *< 0.05) but only slightly reduced MCAO-induced enhanced expression of iNOS and cytokines

**Conclusion:**

The present study shows elevated microvascular expression of TNF-α, IL-1ß, IL-6 and iNOS following focal ischemia, and shows that this expression is transcriptionally regulated via the MEK/ERK pathway.

## Background

Focal cerebral ischemia is a result of reduced cerebral blood flow to a discrete region of the brain, and this initiates a complex process that includes release of excitatory neurotransmitters and activation of apoptotic pathways. Even though regional cerebral blood flow may be restored to near-normal values after 2 hours of middle cerebral artery occlusion (MCAO) by release of the block and consequent reperfusion [1], a cerebral infarct involving about 25% of total brain volume occurs consistently [2]. Some manifestations of the ischemic damage are break-down of the blood-brain barrier, activation of inflammatory cascades, and disruption of basement membranes and extracellular matrix via cytokine-induced alterations in the expression of metalloproteinases [3].

Ischemia initiates a complex process in which inflammation contributes to stroke-related brain injury. This is evident in the systemic circulation as neutrophilia, lymphocytopenia and increased levels of monocytes [4]. There is an early accumulation of neutrophils in the brain, and transmigration of adhesion molecules which are associated with cytokine signaling [5]. In stroke-induced brain injury cytokines such as tumor necrosis factor-α (TNF-α), interleukin-1ß (IL-1ß), interleukin-6 (IL-6), and inducible nitric oxide synthase (iNOS), are produced by a variety of activated cell types; endothelial cells, microglia, neurons, platelets, monocytes, macrophages and fibroblasts [5]. The pattern of cytokine inflammation response differs depending on stroke type and localization. Even though regional cerebral blood flow may be restored to near normal values after MCAO through reperfusion [1], a reproducible cerebral infarct occurs [2]. The ischemic region consists of two parts: the ischemic core and the penumbra, both of which are recognized in clinical practice. Activation of pro-inflammatory cytokines and iNOS in vessel walls after cerebral ischemia may facilitate this process. Thus, neuroinflammation is in principle a defence mechanism designed to neutralize an insult and to restore structure and function of the brain after an insult. Basically, neuroinflammation can be viewed as a protective mechanism that isolates the damaged brain tissue from uninjured areas, destroys affected cells, and repairs the extracellular matrix [6]. All cells in the brain participate in these inflammatory responses, including microglia, macrophages, astrocytes, neurons, and oligodendrocytes. The main mediators of neuroinflammation are glial cells, constituting 70% of the total cell population in the central nervous system. Thus, microglial cells show a rapid response involving cell migration, proliferation, and release of cytokines, chemokines and trophic factors. In addition, there is recruitment of polymorphonuclear leukocytes (PMN) from the circulation. PMN migration involves chemotaxis, adhesion to endothelial cells, penetration of tight junctions and migration through the extracellular matrix [7]. A co-ordinated program of inflammation and resolution initiates in the first few hours after an inflammatory response has begun [8]. In recent years glial cells have received growing attention for their role in coupling events between synaptic activity and glucose metabolism [9,10].

In the nucleus, activation of NF-kB plays a key role [11]; it promotes gene expression and mediates transcription of many genes implicated in the inflammatory response (e. g. TNF-α, IL-1ß, IL-6, iNOS, inter alia) [12]. As outlined in many reviews the neuroinflammation process is complex and involves numerous pathways and molecules in the brain; however, relatively little attention has been directed towards the role of cerebrovascular smooth muscle cells (SMCs) in this process following cerebral ischemia.

Therefore, we aimed to examine early changes in the expression of inflammatory signals that are known to be involved in cerebral ischemia; first; to show enhanced expression of pro-inflammatory mediators (iNOS and cytokines) after transient MCAO in cerebrovascular SMCs, and second, to evaluate if a specific transcription MEK/ERK1/2 inhibitor (U0126) could modify this enhanced expression, and to compare this to a combined inhibition of upregulated angiotensin AT_1 _and endothelin ET_A _receptors.

## Methods

### Middle cerebral artery occlusion

A total of 30 male Wistar-Hanover rats (Møllegaard Breeding Centre, Copenhagen, Denmark), each weighing approximately 300-350 g, were obtained from Harlan, Horst, Netherlands. The animals were housed under controlled temperature and humidity with free access to water and food and were divided in 6 groups comprising both control and treatment groups (5 rats in each group). The experimental procedures were approved by the University Animal Ethics Committee (M43-07).

Anaesthesia was induced using 4.5% halothane in N_2_O:O_2 _(70%:30%) and was maintained by inhalation of 1.5% halothane by mask. To confirm proper occlusion of the right MCA, a laser-Doppler probe (Perimed, Järfälla, Sweden) was fixed to the skull (1 mm posterior to the bregma and 6 mm from the midline on the right side) to measure regional cortical blood flow. A polyethylene catheter was inserted into a tail artery to measure mean arterial blood pressure, pH, pO_2_, pCO_2_, and plasma glucose. A rectal temperature probe connected to a homeothermal blanket was used to maintain body temperature at 37°C during the procedure.

An intraluminal filament technique was used to induce transient MCAO [2]. Briefly, an incision was made in the midline of the neck, and the right common, external, and internal carotid arteries were exposed. The common and external carotid arteries were permanently ligated with sutures. A filament was inserted into the internal carotid artery via an incision in the common carotid artery and advanced until the rounded tip reached the entrance to the right MCA. The resulting occlusion was visualized by laser-Doppler as an abrupt 80-90% reduction in cerebral blood flow. After 2 h of occlusion, the rat was re-anesthetized to allow withdrawal of the filament; reperfusion was verified by laser-Doppler recording.

### Treatments

Experimental treatment regimes were done using two strategies; one to inhibit transcription using a specific inhibitor of MEK/ERK signalling, and the second to inhibit MCAO-induced upregulation of G-protein coupled receptors. Therefore, to inhibit MEK1/2, animals were injected intraperitoneal with 30 mg/kg/day of U0126 dissolved in dimethylsulfoxide (DMSO), beginning at reperfusion (0 h), or at 6 h, 12 h, or 24 h post-occlusion [13]. Rats in the control groups were injected with equal volumes of DMSO. It is well-known that U0126 passes the blood-brain barrier with difficulty. Hence, we chose a rather large dose of U0126 which was chosen on the basis of previous experiments [14].

A second group of animals were given specific receptor blockade by administration intraperitoneal of Candesartan (0.05 mg/kg) and ZD1611 (3-(4-[3-(3-methoxy-5-methylpyrazine-2-ylsulfonyl)-2-pyridyl]-2,2-dimethylpropanoic acid) (0.15 mg/kg) immediately after the occlusion and 24 hours later [13]. This research direction is based on our findings that AT_1 _and ET_A _receptors are crucial for development of the infarct size in this model of cerebral ischemia. For the control group, rats were injected with equal volume of DMSO.

### Harvesting cerebral vessels and brain tissue

At 48 hours post-MCA occlusion, MCAO rats, MCAO rats treated with either U0126 or Candesatan/ZD1611, and their respective DMSO controls were anesthetized and decapitated. The brains were removed and immersed in ice-cold bicarbonate buffer solution of the following composition (mM): NaCl 119, NaHCO_3_, 15, KCl 4.6, MgCl_2 _1.2, NaH_2_PO_4 _1.2, CaCl_2 _1.5, and glucose 5.5. The right and left MCAs and surrounding brain tissue were dissected out using a dissection microscope, snap frozen, and stored at -80°C for immunohistochemical and confocal microscopy analysis.

### Neurological examination

The animals were subjected to a neurological examination prior to recirculation and immediately before they were sacrificed (48 hours after MCAO), according to an established scoring system [15,16] (Table 1).

**Table 1:** Neurological status after MCA occulusion [15,16]

Score	Interpretation
0	No visible deficits
1	Contralateral forelimb flexion, when held by tail
2	Decreased grip of contralateral forelimb
3	Spontaneous movement in all directions, but contralateral circling if pulled by tail
4	Spontaneous contralateral circling
5	Death

### Brain damage evaluation

The brains were sliced coronally in 2-mm-thick slices and stained with 1% 2, 3, 5-triphenyltetrazolium chloride (Sigma, St Louis, MO), dissolved in buffer solution at 37°C, for 20 minutes. The extent of ischemic brain damage was calculated as a percentage of the total brain volume in the slices using the software program Brain Damage Calculator 1.1 (MB Teknikkonsult, Lund, Sweden).

### Immunofluorescence

For immunofluorescence analysis, the MCA and the surrounding brain tissue were dissected out, placed into Tissue TEK (Gibo, Invitrogen A/S, Taastrup, Denmark), and frozen on dry ice; thereafter, they were sectioned into 10-μm-thick slices. Cryostat sections of the arteries and brain tissue were fixed for 10 minutes in ice-cold acetone (-20°C) and then rehydrated in phosphate buffer solution (PBS) containing 0.3% Triton X-100 for 15 minutes. The tissues were then permeabilized and blocked for 1 hour in blocking solution containing PBS, 0.3% Triton X-100, 1% bovine serum albumin (BSA), and 5% normal donkey serum, and then incubated overnight at 4°C with the following primary antibodies: rabbit anti-rat iNOS (Abcam, ab15326), rabbit anti-rat IL-6 (Abcam, ab 6672), rabbit anti-rat IL-1ß (Abcam, ab9787) diluted 1:400, rabbit anti-rat TNF-α (18285; IBL, Japan) diluted 1:20, rabbit anti-phospho p38 (Cellsignalling, #9212), rabbit anti-phospho ERK1/2 MAPK (Cellsignalling, #4376) diluted 1:50 and rabbit anti-phospho SAPK/JNK (Cellsignalling, #.9251) diluted 1:100. All primary antibodies were diluted in PBS containing 0.3% Triton X-100, 1% BSA, and 2% normal donkey serum. Sections were subsequently incubated for 1 hour at room-temperature with secondary Cy™²-conjugated donkey anti-rabbit (711-165-152; Jackson ImmunoResearch, Europe Ltd., Suffolk, UK) diluted 1:200 in PBS containing 0.3% Triton X-100 and 1% BSA. The sections were subsequently washed with PBS and mounted with Permafluore mounting medium (Beckman Coulter, PNJM0752). Immunoreactivity was visualized and photographed using a Nikon confocal microscope (EZ-c1, German) at the appropriate wavelength. The same procedure was used for the negative controls except that primary antibodies were omitted.

### Double immunofluorescence

Double immunofluorescence labelling was performed for IL-1 ß, IL-6, iNOS, TNF-α, and phosphorylated ERK1/2 versus smooth muscle actin, expressed in the smooth muscle cells. In addition to the antibodies described above, we used mouse anti-rat smooth muscle actin antibodies (SC-53015; Santa Cruz Biotechnology, Inc, Santa Cruz, CA) diluted 1:200 in PBS containing 0.3% Triton X-100, 1% BSA, and 2% normal donkey serum. The secondary antibodies were Cy™²-conjugated donkey anti-rabbit (Jackson ImmunoResearch) diluted 1:200 and Texas Red-labeled donkey anti-mouse (Jackson ImmunoResearch Europe) diluted 1:300 in PBS containing 0.3% Triton X-100 and 1% BSA. The antibodies were detected at the appropriate wavelengths using a Nikon confocal microscope (EZ-cl, Germany).

### Image analysis and protein measurement for immunoflourescence

Fluorescence intensity shown was related to expression of protein levels in each sample and measured with a semiquantitative method. Fluorescence intensity was measured using ImageJ software http://rsb.info.nih.gov/ij/. Measurements were made in 4 to 6 different areas (located on the clock at 0, 3, 6 and 9 h) for each tissue sample. The investigator was blinded to the treatment group of each sample, the fluorescence intensity of each group was given as the percentage change relative the control and the control value was normalized to 100%. The mean value for each was used for comparisons [13,17].

### Calculations and statistical analyses

Data are expressed as mean ± standard error of the mean (s.e.m). Statistical analyses were performed using the nonparametric Kruskal-Wallis test with Dunn's post hoc test. *P*-values less than 0.05 were considered significant; "n" refers to the number of rats.

## Results

**Figure 1:**
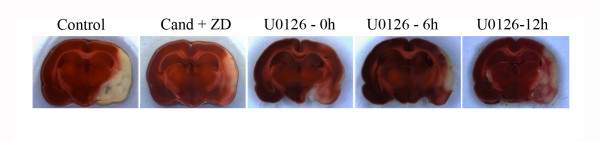
**Typical examples of coronal brain sections that show smaller ischemic areas in rats treated with combined Candesartan + ZD1611, U0126 given at 0 and 6 hours after MCAO versus animals treated with vehicle (control)**. Treatment with U0126 given at 12 hours after MCAO did not decrease the ischemic area.

**Figure 2:**
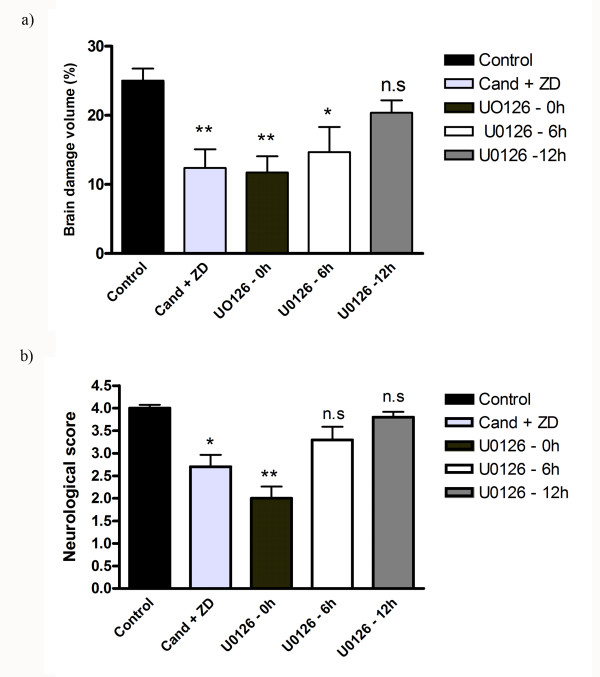
a): Size of infarct volume (% of total brain volume) was significantly decreased in middle cerebral artery occlusion (MCAO) animals treated with U0126 starting at 0 hours (11.7 ± 2%**) and 6 hours (14.6 ± 3%*) after MCAO as compared to the control group (25 ± 2%). Treatment with U0126 starting at 12 hours after MCAO did not significant decrease the area of ischemia (20.3 ± 1%). Treatment with combined inhibition of angiotensin AT_1 _(Candesartan) and endothelin ET_A _(ZD1611) receptors significantly decreased brain damage (12.3 ± 3). Data are expressed as mean ± SEM; n = 4-7. *P < 0.05, ** < 0.01. **b): **Neurological scores in MCAO rats were significantly improved with combined candesartan + ZD1611 and U0126 starting at 0 hours (2.6 ± 0.7% and 2 ± 0.7, respectively), compared to vehicle-treated rats (control). Data are expressed as mean ± SEM; n = 6-7. *P < 0.05, ** *P *< 0.01.

In this study, we used a rat model of inducible cerebral ischemia: animals were subjected to reversible MCAO for 2 hours followed by reperfusion for 48 hours [2]. About 20 - 25% of animals in each group were excluded from data analysis due to failure during the operational procedure (the drop of cortical blood flow was too small or technical problems). Immediately before MCAO, physiological parameters (blood pressure, blood gases, temperature, plasma glucose, and body weight) were measured and there were no significant differences between the different treatment groups (data not shown) MCAO produced an occlusion visible by laser Doppler flowmetry as an abrupt 80 - 90% reduction in cerebral blood flow that normalized after removal of the occluding thread. Following rapid sacrifice, tissues were collected for immunocytochemistry and calculation of infarct volume (25 ± 2% of total cerebrum in the MCAO group; Figures 1 and 2a). Neurological evaluations were performed just before animal sacrifice (MCAO group, 4.0 ± 0.2 versus sham-operated animals with no visible defects resulting in a score of 0; *P *< 0.05, Figure 2b).

### Analysis of infarct volume and neurological examination

Previously, immunocytochemical and western blot analysis showed that MCAO with reperfusion causes activation of the MEK/ERK pathway and endothelin and angiotensin receptor upregulation in cerebral vessels associated with the ischemic region [14]; data from the present study confirms this observation. Firstly, intravenous administration of the MEK1/2 inhibitor U0126 [18] starting at 0 or 6 hours after MCAO and reperfusion significantly reduced infarct volume (11.7 ± 2% and 15 ± 3%, respectively; *P *< 0.05; Figures 1 and 2a) and improved neurological assessment scores (2 ± 0.7 and 3.3 ± 0.7, respectively; *P *< 0.05, Figure 2b). When U0126 treatment was initiated 12 hours after the start of reperfusion, there was no significant reduction in infarct volume or neurological score when compared to control animals (21 ± 2% and 3.8 ± 0.8%, respectively; *P *< 0.05; Figures 1, 2a and 2b). Secondly, analysis of the brains after staining with TTC revealed that the combined treatment with Candesartan and ZD1611, injected immediately after occlusion, resulted in significant reduction in infarct volume (12.3 ± 3%; P < 0.05; Figures 1 and 2a) and improved neurological score at 48 h after occlusion (2.6 ± 0.7%; P < 0.05; Figure 2b).

### Expression and localization of iNOS, IL-1ß, IL-6 and TNF-α

**Figure 3:**
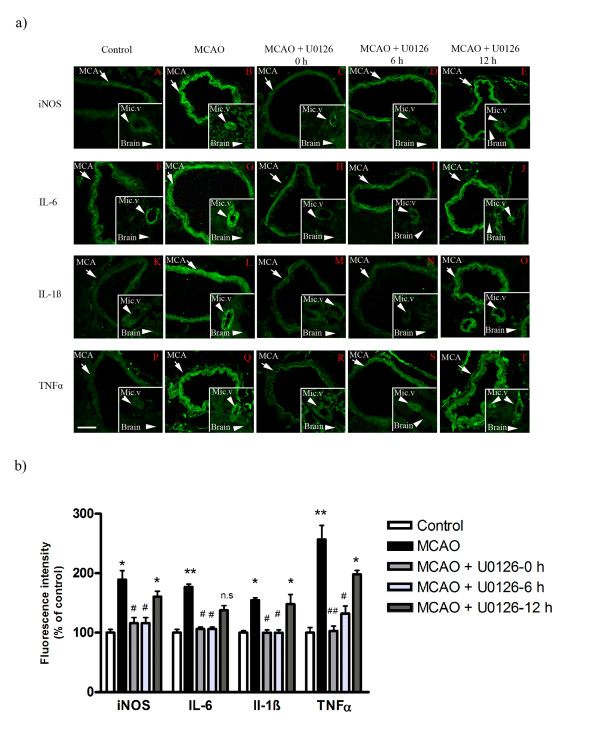
**a): Confocal microscopy images of ischemic middle cerebral artery (MCA), cerebral microvessels (Mic.V), and surrounding brain tissue (Brain) immunofluorescently labeled for iNOS (A-E), IL-6 (F-J), IL-1ß (K-O) and TNF-α (P-T)**. Images represent the vehicle control group (**A, P**), MCAO plus vehicle group (**B, Q**), MCAO plus U0126 starting at 0 hours (**C, R**), 6 hours (**D, S**), or 12 hours (**E, T**). There were significant increases in iNOS, IL-6, IL-1ß and TNF-α protein levels in the smooth muscle cell layer of ischemic vessels (MCA and Mic.V) as compared with vessels from the vehicle control group. Treatment with U0126 starting at zero and 6 hours, but not 12 hours, after occlusion prevented the increase in expression of these proteins. There was a slight increase in iNOS protein expression in ischemic brain tissue around vessels as compared to control and U0126-treated brain tissue. Scale bar, 50 μm. **b): **Bar graphs showing semi-quantification of fluorescence intensity for iNOS, IL-6, IL-1ß and TNF-α in MCA. There were significant increases in the expression of these proteins in MCAO animals as compared to control animals; this increase was prevented with U0126 treatment starting at zero and 6 hours, but not 12 hours, post MCAO. Data are presented as the mean percentage relative to control ± SEM.; *n *= 5. *P < 0.05, significant difference between control and treated at 12 h, **P < 0.01 significant difference between control groups and MCAO, #*P *< 0.05 and ##*P *< 0.01 significant difference between treatment groups (0 h and 6 h) and MCAO groups.

Subsequently, we examined the MCA, cerebral microvessels, and the surrounding brain tissue for changes in protein expression of iNOS and pro-inflammatory cytokines both in the ischemic region and in the contralateral side at 48 hours after MCAO. There was locally enhanced expression of iNOS, IL-16, IL-1ß and TNF-α in smooth muscle cells in the ischemic region both in the MCA leading to the stroke region and in microvasculature walls (Figure 3, Tables 2 and 3). Notably this enhanced expression was primarily seen in smooth muscle cells, while a weak expression occurred in endothelial cells for IL-6 (see below). The surrounding brain tissue (neurons, astrocytes) was only faintly stained as compared with that of the contralateral side for iNOS, IL-6 and IL-1ß, but there was no staining for TNF-α There was a marked expression of iNOS, IL-1ß, IL-6 in vascular smooth muscle cells from the ischemic region, localized to sarcoplasm and leaving the nuclear region clear of expression (Figure 3). Quantification of this expression revealed significant upregulation (Figure 3b, Tables 2 and 3) There was no significant alteration in iNOS, IL-1ß, IL-6 and TNF-α activity in brain tissue of the ischemic or of contralateral regions (Table 4).

**Table 2:** Protein levels of iNOS, IL-6, IL-1ß and TNF-α in MCA after MCAO and treatment with U0126

Middle cerebral artery					
	**Control**	**MCAO**	**MCAO + U0126-0 h**	**MCAO + U0126-6 h**	**MCAO + U0126-12 h**
iNOS (%) ± s.e.m	100 ± 5	185 ± 16*	106 ± 9^#^	116 ± 9^#^	160 ± 9*
IL-6 (%) ± s.e.m	100 ± 6	177 ± 6**	113 ± 5^#^	110 ± 5^#^	137 ± 7
IL-1ß (%) ± s.e.m	100 ± 4	155 ± 4*	112 ± 8^#^	109 ± 4^#^	148 ± 16*
TNF-α ± s.e.m	100 ± 8.5	256 ± 24**	103 ± 8^##^	132 ± 12^#^	198 ± 7*

Values are expressed as percentage of control and given as mean ± s.e.m, n = 5-6, * P < 0, 05, ** P < 0, 01, ^# ^P < 0, 05, ^## ^P < 0, 01. * = significant difference between control groups and MCAO or control and treated at 12 hours, ^# ^= significant difference between MCAO and treatment groups (starting at 0 and 6 h).

**Table 3:** Protein levels of iNOS, IL-6, IL-1ß and TNF-α in microvessels after MCAO and treatment with U0126

Micro-vessels					
	**Control**	**MCAO**	**MCAO + U0126-0 h**	**MCAO + U0126-6 h**	**MCAO + U0126-12 h**
iNOS (%) ± s.e.m	100 ± 7.2	144 ± 1.0*	95 ± 10^#^	109 ± 3.01	112 ± 8.4
IL-6 (%) ± s.e.m	100 ± 8.6	134 ± 4.4*	106 ± 7.8^#^	101 ± 2.7^#^	119 ± 3.6
IL-1ß (%) ± s.e.m	100 ± 3.1	125 ± 4.9*	113 ± 5.5^#^	96 ± 2.4^#^	122 ± 14.2
TNF-α ± s.e.m	100 ± 7.1	205 ± 12*	117 ± 11^#^	117 ± 7.1^#^	149 ± 9*

Values are expressed as percentage of control and given as mean ± s.e.m, n = 5-6,

*and ^# ^P < 0, 05, * = significant difference between control groups and MCAO or control and treated at 12 hours, ^# ^= significant difference between MCAO and treatment groups (starting at 0 and 6 hours).

**Table 4:** Protein levels of iNOS, IL-6, IL-1ß and TNF-α in brain tissue after MCAO and treatment with U0126

Brain-tissue					
	**Control**	**MCAO**	**MCAO + U0126-0 h**	**MCAO + U0126-6 h**	**MCAO + U0126-12 h**
iNOS (%) ± s.e.m	100 ± 7	112.8 ± 6	104 ± 3	100.2 ± 5	103 ± 9
IL-6 (%) ± s.e.m	100 ± 4	108 ± 3.4	94 ± 5	95 ± 3.7	102 ± 5.6
IL-1ß (%) ± s.e.m	100 ± 4.7	122 ± 8.6	100 ± 5	100 ± 3	111 ± 3.1
TNF-α ± s.e.m	100 ± 8	119 ± 14	117 ± 10	114 ± 7.6	122 ± 8.9

Values are expressed as percentage of control and given as mean ± s.e.m, n = 5-6,

### Inhibition AT_1 _and ET_A _receptors in vivo

**Figure 4:**
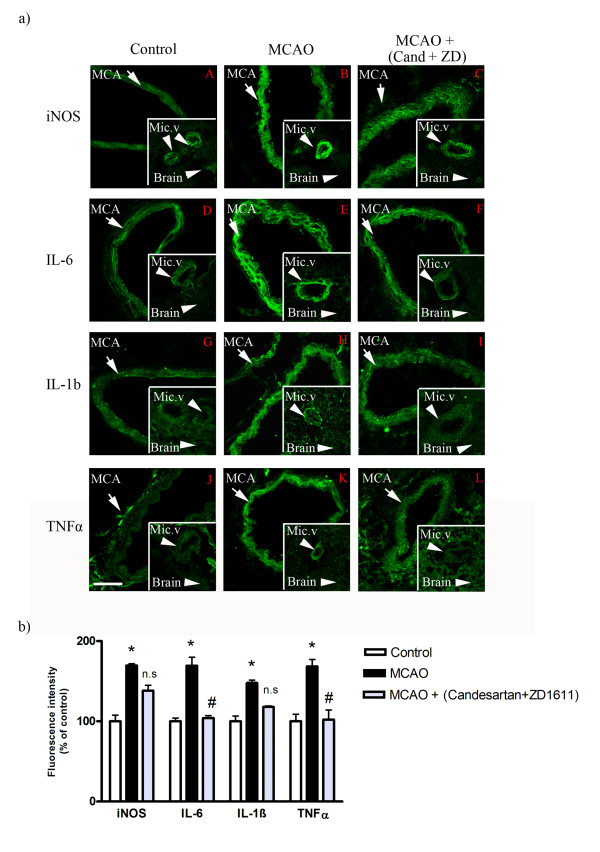
**a): Sections from the middle cerebral artery (MCA), cerebral microvessels (Mic.V), and surrounding brain tissue (Brain) showing iNOS, IL-6, IL-1ß and TNF-α immunoreactivity in the smooth muscle cell layer**. A) iNOS; control, B) iNOS; MCAO, C) iNOS; MCAO treated with AT_1_/ET_A _receptor inhibitor (candesartan + ZD1611), D) IL-6;control, E) IL-6; MCAO, F) IL-6; MCAO treated with candesartan + ZD1611, G) IL-1 ß; control, H) IL-1 ß; MCAO, I) IL-1 ß; MCAO treated with candesartan + ZD1611, J) TNF-α; control, K) TNFα; MCAO, L) TNFα, MCAO treated with candesartan + ZD1611. There were increased expressions of iNOS, IL-6, IL-1ß and TNF-α protein levels in both middle cerebral artery and cerebral microvessels after MCAO as compared to control groups. Treatment with AT1/ETA receptor inhibitors starting immediately after induced MCAO diminished the increases in protein levels. There was no change in protein expression in ischemic brain tissue around vessels as compared to control and treated brain tissue. Data were obtained with confocal microscopy. Scale bar 50 μm. **b): **Bar graphs showing fluorescence intensity for iNOS, IL-6, IL-1ß and TNF-α in MCA. There were significant increases of the espressions of these proteins in MCAO animals as compared to control animals. Treatment with AT_1_/ET_A _receptor inhibitors starting at 0 hours produced significant decreases in IL-6 and TNF-α protein levels and slight decreases in iNOS and IL-1ß protein levels. Data are presented as the mean percentage relative to control ± SEM.; *n *= 5. *P < 0.05, significant difference between control group and MCAO, #*P *< 0.05, significant difference between MCAO and treated group.

In a previous study we observed that endothelin and angiotensin receptors were upregulated in cerebrovascular smooth muscle cells [19,20]; here we tested the hypothesis that these receptors might be involved in the upregulation of cytokines and iNOS activity in vessels walls. Thus, the administered combination of the angiotensin AT_1 _blocker Candesartan and the endothelin-1 ET_A _blocker ZD1611 systemically resulted in reduced infarct size and in a better neurological score after ischemic stroke in rat but not in enhanced receptor expression induced by MCAO [13]. According to other studies, both angiotensin II and endothelin-1 are pro-inflammatory and these have previously been found to be increased in cerebral ischemia. Here we found that specific receptor antagonism reduces infarct volume and neurological score (Figures 1, 2a and 2b), and in part the elevated expression of IL-1 ß, IL-6, TNF-α and iNOS (Figure 4 and Table 5).

**Table 5:** Protein levels of iNOS, IL-6, IL-1ß and TNF-α after MCAO and treatment with a combination of AT_1_/ET_A _receptor antagonists

	Middle cerebral artery			Micro-vessels			Brain tissue		
	**Control**	**MCAO**	**Treated**	**Control**	**MCAO**	**Treated**	**Control**	**MCAO**	**Treated**
iNOS(%) ± s.e.m	100 ± 5.4	168 ± 7*	139 ± 7.7	100 ± 11.5	146 ± 10*	118 ± 13	100 ± 5	118 ± 9	106 ± 6
IL-6(%) ± s.e.m	100 ± 3	180 ± 13*	104 ± 3^#^	100 ± 4.9	146 ± 17*	103 ± 4	100 ± 8.9	109 ± 13	101 ± 12.2
IL-1ß(%) ± s.e.m	100 ± 5	146 ± 3*	117 ± 4.5	100 ± 5	117 ± 3.6	101 ± 3.8	100 ± 0.6	113 ± 4	102 ± 6.4
TNF-α ± s.e.m	100 ± 8	168 ± 8.7*	102 ± 12^#^	100 ± 5	117 ± 3.6	101 ± 3.8	100 ± 0.6	113 ± 4	102 ± 6.4

Values are expressed as percentage of control and given as mean ± s.e.m, n = 3-4, ^#,^* P < 0, 05, * significant difference between control groups and MCAO, ^# ^= significant difference between MCAO and treatment groups.

### Enhanced expression of MAPK and inhibition of MEK1 activity in vivo

**Figure 5:**
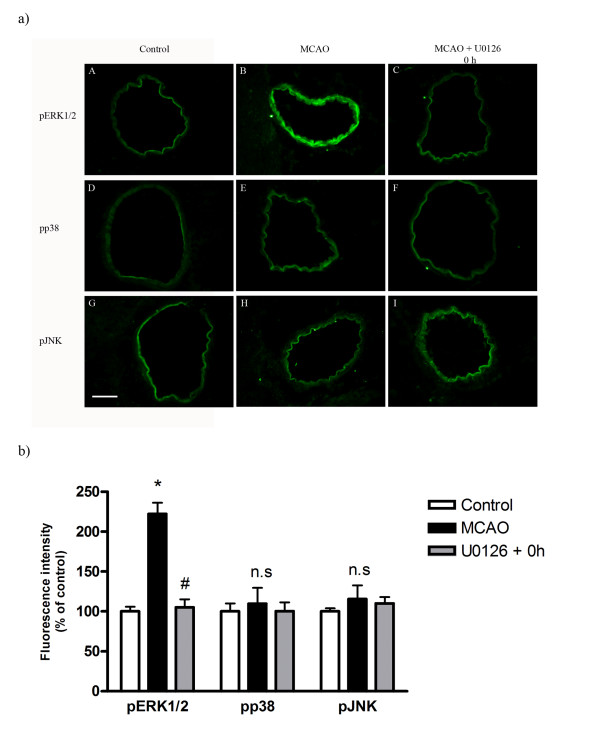
**a): Confocal microscopy images of ischemic middle cerebral artery (MCA) labeled for pERK 1/2 (A-C), pp38 (D-F) and pJNK (G-I)**. There was increased pERK 1/2 in MCA of the MCAO group as compared with control, and treatment with U0126 at 0 hour prevented this increase. Activation of pp38 and pJNK were not changed in the MCAO and treatment groups. Scale bar 50 μm. **b): **Bar graphs showing fluorescence intensity for pERK1/2, pp38 and pJNK in MCA. Activation of pERK1/2 was significantly increased in MCAO as compared to control group. Treatment with U0126 significantly decreased this activation. There was no alteration in protein expression of pp38 and pJNK in the control, MCAO and treated groups. Data are presented as the mean percentage relative to control ± SEM.; *n *= 5. *P < 0.05, significant difference between control group and MCAO, #*P *< 0.05, significant difference between MCAO and treated group.

We subsequently assessed if there were activation of MAPK in the walls of the MCA, the microvessels and surrounding brain tissue after MCAO. At baseline only a faint expression was observed of pERK1/2 in the cerebral vessel walls. After MCAO, pERK1/2 activity in vascular smooth muscle cells was significantly upregulated in large cerebral arteries (185 ± 11%; *P *< 0.05) and in microvessels (130 ± 10%; *P *< 0.05) (Figure 5) but not in adjacent brain tissue (103 ± 4%; *P *> 0.05). U0126 treatment initiated at time zero hours after initiation of reperfusion normalized vascular pERK1/2 expression (108 ± 5%, *P *< 0.05, Figure 5). Results from double immunostaining with pERK1/2 and actin localized this enhanced expression to smooth muscle cells (data not shown). There was no or only very weak expression of pp38 and pJNK in baseline samples and these were not changed after MCAO and treatment with U0126 starting at 0 hour (with injection) (110 ± 20%, 115 ± 17%, respectively; *P *< 0.05; Figures 5a and 5b).

Because of the data from the MAPK analysis we focused on the MEK/ERK1/2 pathway. Systemic administration of the potent MEK1-specific inhibitor U0126, which blocks the enzymatic activity of MEK1, starting either immediately after occlusion (0 hour) or at 6 hours after MCAO, effectively abolished the increase in pERK1/2 activity in ischemic MCA and in cerebral microvessels. There was no alteration in pERK1/2 activity in brain tissue of the ischemic or of contralateral regions.

**Figure 6:**
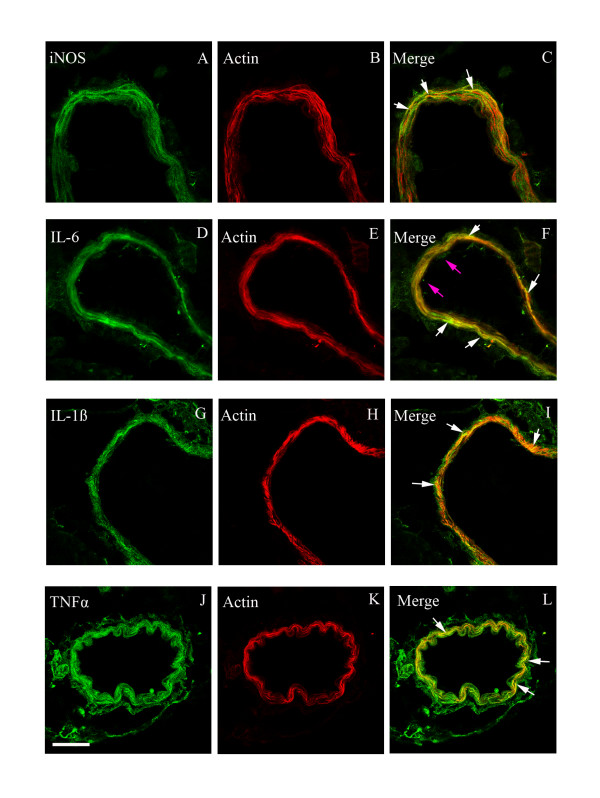
**Double immunofluorescent staining for iNOS, IL-6, IL-1ß, TNF-α and actin in smooth muscle cells of the middle cerebral artery after MCAO**. (**A**-**C**) Photographs demonstrating the localization of iNOS and actin immunostaining, and their co-localization in smooth muscle cells (yellow fluorescence in the merged picture). (**D**-**F**) IL-6 and actin immunostaining, and their co-localization in smooth muscle cells (white arrows). (G-I) IL-1ß and actin immunostaining, and their co-localization (yellow fluorescence in the merged picture) in smooth muscle cells. (J-L) TNF-α and actin immunostaining, and their co-localization in smooth muscle cells (yellow fluorescence in the merged picture). Enhanced expressions of iNOS, IL-1ß and TNF-α proteins are located in smooth muscle cells while IL-6 is located in both smooth muscle (white arrows) and endothelial cells (pink arrows). Scale bar, 50 μm.

We observed that treatment with U0126 (with start at 0 or 6 hours) resulted in a decrease of the upregulated activity of iNOS, IL-1ß, IL-6 and TNF-α in both the MCA and the cerebral microvessels within the infarct area (Figure 3, Tables 2 and 3). According to the co-localization with actin in the smooth muscle cells, their expression was localized to the vascular smooth muscle cells, localized to the cytoplasm and leaving the nuclear region clear of expression; for IL-6 we observed also some staining of the endothelial cells (Figure 6). However, administration of U0126 with a start 12 hours after MCAO did not significantly reduce the ischemia induced expression of iNOS, IL-1ß, IL-6 or TNF-α in the cerebral vessel smooth muscle cells (Figure 3, Tables 2 and 3).

## Discussion

This is the first study to clearly demonstrate that MCAO for 2 hours and reperfusion for 48 hours resultes in significant upregulation of iNOS, IL-1ß, IL-6 and TNF-α in smooth muscle cells of the MCA and of microvessels within the ischemic region. Furthermore, our experiments revealed that this upregulation is probably a transcriptional event because it is associated with a parallel upregulation of pERK1/2, and these changes are all normalized by inhibition with U0126.

The specific MEK1 inhibitor U0126 has been shown not to affect phosphorylation of p38 or JNK in cultured neurons [21] or in cerebrovascular smooth muscle cells *in vivo *[22]. Therefore, we can exclude the possibility that U0126 acts via non-specific inhibition of pro-apoptotic and pro-inflammatory mechanisms. U0126 has been found to increase MEK1/2 phosphorylation in cortical neurons, hence U0126 does not affect upstream components of MEK1/2 [23]. Thus, it is reasonable to assume that the neuroprotective effect of U0126 is the result of inhibition of cerebrovascular MEK1 activity, which is in agreement with reductions in the activity of the downstream MAPK pERK1/2. Presently, MCAO results in enhanced expression of pERK1/2 in smooth muscle cells of ischemic MCA and in associated microvessels (shown by co-localization with actin) but not in surrounding brain tissue; U0126 blunts this activation in parallel with a reduction in infarct volume and an improved neurological score. The other MAPK p38 and JNK were only mildly affected in vessel walls by MCAO; however, as shown before there is enhanced pp38 and pJNK in brain tissue subsequent to stroke that is mainly localized to neurons and glial cells, and this occurs late in the process [24].

Quite intriguing is our observation that inhibition of this sequence of events correlates with inhibition of iNOS, IL-1ß, IL-6 and TNF-α expression in the same locale. Quantitative real-time PCR has demonstrated enhanced mRNA expression of iNOS, IL-1ß and IL-6 at 24 hours after MCAO [22]. Our data suggest for the first time that the enhanced expression of iNOS, IL-1ß, IL-6 and TNF-α in cerebral ischemia is a transcription/translational event in brain vessels, and points to a way to modify their expression by MEK/ERK1/2 inhibition.

Previous work has indicated that MEK/ERK1/2 mechanisms play a crucial role in brain injury after ischemia and reperfusion, with reductions in infarct size resulting from inhibition of these mechanisms [21,25]. Here, we provide direct evidence for a possible explaination for some of the events that are related to the focal pathology of cerebral ischemia. U0126 diminishes pERK1/2 immunoreactivity in ischemic brain of mice [21] and in the MCA of rats. In a mouse model employing MCAO for 3 hours followed by reperfusion for 24 hours, infarct volume was only affected if U0126 was given at the time of MCAO [21]; this might, however, be due to the use of too low a dosage. In a model using permanent MCAO, pretreatment with U0126 was necessary to inhibit pMEK1/2 and pERK1/2 expression in the neuropil. Also the specificity of antagonisms revealed that U0126 does not inhibit the cellular synthesis of ERK1/2 but blocks ERK1/2 phosphorylation and activation of molecules such as transcription factor ELK-1. In agreement with our observations, MEK1 inhibitors have been found to not alter cortical blood flow in the first few hours of administration [21,26], nor do they modify the contractility of isolated cerebral arteries (Maddahi and Edvinsson, unpublished data). Hence, MEK1 is not acting on the cerebral circulation via a direct vasodilator mechanism but, as we suggest, blunts receptor upregulation [25]. We have here demonstrated yet another important mechanism involved in cerebral ischemia, neuroinflammation, and we have demonstrated that U0126 blocks enhanced cerebrovascular expression of iNOS, IL-1ß, IL-6 and TNF-α.

Following cerebral ischemia there is a pro-inflammatory response, with infiltration of cells and generation of interleukins and iNOS, often seen after a delay of about 24 hours following experimental focal ischemia. In this study, we observe for the first time that, in conjunction with MCAO and reperfusion for 48 hours, there is an elevated expression of iNOS, IL-1ß, IL-6 and TNF-α in MCA and in cerebral microvessels within the ischemic region. This expression is localized to smooth muscle cells (co-localization with actin). At a time point 24 hours after MCAO, real time-PCR has demonstrated within vessel walls enhanced expression of mRNA for IL-1ß and IL-6 but not for iNOS [22]. This supports involvement of a transcriptional event. Wang and colleagues observed that the level of IL-1ß is increased in ischemic regions after permanent MCAO in mice [27,28] and [29]. This enhanced expression of IL-1ß mRNA is inhibited by U0126 treatment. Interestingly, protein expression in these smooth muscle cells appears to be upregulated via *de novo *transcription involving the MEK/ERK1/2 pathway since inhibition of pERK1/2 activity with MEK1 inhibitor markedly reduces their expression.

Endothelin-1 and angiotensin II are involved in inflammatory processes, in addition to their potent vasomotor effects; consequently direct receptor inhibition might be a good way to reduce infarct volume after MCAO. The use of specific endothelin [30] or angiotensin receptor antagonists [31] has been found to provide some reduction in infarct size, but a combined receptor blockade shows stronger effects due to combined upregulation of several receptor subtypes [13]. However, such combined treatment does not modify the upregulation of the G-protein coupled receptors studied, but blocks their effect and reduces infarct size. However, while receptor antagonism does not change the upregulation of pERK1/2 and iNOS levels, it does reduce the expression of IL-1ß, IL-6 and TNF-α in vessel walls, possibly via an anti-inflammatory effect. The background to the use of the present receptor blockade was a series of studies on receptor regulation in cerebral ischemia models which has revealed elevation of AT_1_, ET_A _and ET_B _receptors [19,20] and [32] in MCA smooth muscle cells. Our decision not to choose an ET_B _receptor blocker was based on negative experimental data [33], on the fact that upregulation of contractile ET_B _receptors takes some time [34,35], and on the fact that early treatment results only in blockade of vasodilator ET_B _receptors (which may be of value during early phases of stroke).

## Conclusion

The use of MEK1 inhibition might be a way to obtain anti-stroke efficiency since it targets several transcriptional mechanisms activated by cerebral ischemia: (i) receptor upregulation causing enhanced contractility, and (ii) inflammatory gene activation, i.e., for iNOS, IL-1ß, IL-6 and TNF-α. MEK1 inhibition, applied as late as 6 hours after the start of reperfusion, significantly reduced infarct volume and, in parallel, reduced the upregulation of contractile receptors, and reduced the elevation of iNOS, IL-1ß, IL-6 and TNF-α in cerebral vessel walls. The positive effects of a MEK1 inhibitor might thus involve several mechanisms in MCA and in brain microvasculature associated with the cerebral ischemia.

## Competing interests

The authors declare that they have no competing interests.

## Authors' contributions

AM carried out all the experiments, participated in the design, statistical analysis and writing of the manuscript. LE conceived the study and the design, coordinated the work and the writing of the manuscript. Both authors have read and approved the final manuscript.
